# Gut bacteria presence in the brain is increased after ischemic stroke in mice

**DOI:** 10.1080/19490976.2026.2617694

**Published:** 2026-01-26

**Authors:** Alex Peh, Evany Dinakis, Michael Nakai, Rikeish R. Muralitharan, Samoda Rupasinghe, Jenny L. Wilson, Connie H.Y. Wong, Hamdi A. Jama, Charlotte M.O. Barker, Mahnaz Modarresi, Barbara K. Kemp-Harper, Tenghao Zheng, Francine Z. Marques, Brad R.S. Broughton

**Affiliations:** a Hypertension Research Laboratory, School of Biological Sciences, Monash University, Melbourne, Australia; b Cardiovascular & Pulmonary Pharmacology Group, Department of Pharmacology, Monash University, Melbourne, Australia; c Institute for Medical Research, Ministry of Health Malaysia, Kuala Lumpur, Malaysia; d Centre for Inflammatory Diseases, Department of Medicine, School of Clinical Sciences at Monash Health, Monash Medical Centre, Clayton, Victoria, Australia; e Heart Failure Research Group, Baker Heart and Diabetes Institute, Melbourne, Australia

**Keywords:** Stroke, gut microbiome, dysbiosis, sympathetic nervous system

## Abstract

Systemic infections are a common cause of complications and death after stroke. These infections can occur due to the breakdown of the gut epithelial barrier and the translocation of bacteria from the gut to peripheral systemic tissues. However, it remains unclear whether gut bacteria also translocate to the brain and contribute to stroke-induced neuronal damage. In this study, we observed a significant number of peptidoglycan- and lipopolysaccharide-positive bacteria in the ischemic hemisphere of mice subjected to either photothrombotic (PT) stroke or middle cerebral artery occlusion (MCAO). In contrast, no bacteria were observed in the ischemic brains of germ-free mice following MCAO. Absolute quantification via PCR also revealed increased bacteria in the ischemic hemisphere and blood of PT mice. Bacterial translocation to the brain is associated with the breakdown of the gut-epithelial and blood–brain barriers. Although inhibition of sympathetic tone reduces gut–epithelial barrier permeability, the bacterial load in the brain and functional deficits poststroke, it does not affect cerebral apoptosis, neuroinflammation or infarct volume. Collectively, these findings indicate that activation of the sympathetic nervous system after stroke promotes the migration of gut-derived bacteria into the ischemic brain, and this process worsens motor function in mice.

## GRAPHICAL ABSTRACT

**Figure:**
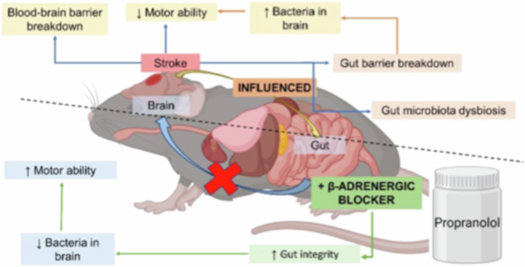

## Introduction

Stroke is the leading cause of disability in high-income countries such as Australia, England, and the United States, and the second leading cause of death globally.^1^ After a stroke, the gastrointestinal (GI) system, regulated by both the central and peripheral nervous systems, undergoes diverse functional alterations. The clinical incidence of dysphagia,^2^ GI bleeding, delayed GI emptying, and colorectal dysfunction^3^ are often observed in stroke patients and may represent a poor prognosis.^4^ Infection is the most frequent complication of stroke, affecting approximately 30% of patients,^1^ with pneumonia and urinary tract infections being the most common.^5^ Bacterial infections after stroke are associated with neurological sequelae and prolonged hospital stays and are a significant cause of early- and long-term morbidity and mortality.^6^^,^^7^ Emerging evidence suggests that bacteria causing lung infection poststroke may originate from the host’s gut.^8^ Such studies report that ischemic stroke disrupts the gut epithelial barrier and reduces gut bacterial diversity.^8^ Thus, the breakdown of the gut epithelial barrier after stroke likely contributes to the high incidence of poststroke infections, with the translocation of bacteria from the gut to other systemic tissues, such as the lungs.^8^

However, there is no evidence of whether gut bacteria migrate to the brain poststroke. While the brain–blood barrier (BBB) prevents bacteria from entering the brain under healthy conditions, it is severely compromised in the ischemic hemisphere after stroke.^9^ Thus, we hypothesized that both the BBB and the gut–epithelial barrier are dysfunctional following stroke, leading to the translocation of commensal gut bacteria into the circulation and consequently into the brain. In this study, we aimed to determine the presence of bacteria in the brain and provide a comprehensive characterization of acute intestinal changes poststroke. Using two different experimental mouse models of stroke, we show that bacteria are present in the ischemic brain. This is likely due to the breakdown of the BBB and gut‒epithelial barrier, which is mediated by sympathetic activation in the gut.^9^

## Materials and methods

### Experimental studies

All the experimental procedures performed in this study were approved by the Monash Animal Ethics Committee (animal ethics approval numbers: MARP/2017/076, 23064, and MMCB/2018/002) and followed the Australian Code for the Care and Use of Animals for Scientific Purposes. Male C57BL6/J mice (8‒9 weeks; *n* = 4‒12/group) were purchased from the Monash Animal Research Platform (MARP, Melbourne, Australia). Simple randomization was used to group the mice into naïve, sham or stroke surgery groups. Researchers were blinded to the sample identification by using a unique sample ID for each animal. No mice were excluded from this study. All the measurements were taken from distinct samples.

### Ischemic stroke models

The photothrombotic (PT) and permanent middle cerebral artery occlusion (pMCAO) stroke models were used in this study. PT stroke surgery was performed as previously described.^10^^,^^11^ Briefly, the mice were anesthetized with isoflurane and placed into a stereotaxic frame. We prepared a 10 mg/mL Rose Bengal solution and administered 200 µL of this solution to each mouse via intraperitoneal injection. For a typical 25 g mouse, this corresponds to a final dose of approximately 80 mg/kg body weight. To induce photothrombosis, the skull was exposed, and the bregma was identified as the reference point (anterior–posterior: 0.0 mm, mediolateral: 0.0 mm, dorsoventral: 0.0 mm). The illumination site was positioned 1.5 mm lateral to the right of the bregma (mediolateral: + 1.5 mm) over the M1 motor cortex. The area was illuminated for 15 minutes using a cold LED light source (KL 1600 LED, Schott, Mainz, Germany; luminous flux: 680 lm; color temperature: 5600 K) to activate the dye. The light was focused through a 25 × microscope objective (Zeiss) mounted at the end of the light to ensure precise and localized illumination. The mice were placed on a heating pad throughout the surgery, and their body temperature was monitored via a rectal probe and maintained at 37°C. The presence of bacteria in the brain was examined at 24 and 72 hours after PT stroke. To confirm the presence of bacteria in the brain of another stroke model, conventional and germ-free mice subjected to sham or pMCAO were used, as described previously.^12^ To induce pMCAO, a 10 mm incision was made on the right side of the neck after the mice were anesthetized. A silicon-coated monofilament with a diameter of 0.21–0.23 mm was then inserted into the stump of the external carotid artery and ligated. The wounds in the necks of the mice were sutured, and the mice were transferred to a heating pad to maintain their body temperature at 37°C for recovery. To examine whether stroke-induced changes to the gastrointestinal (GI) tract occur via sympathetic signaling mechanisms, PT mice were injected intraperitoneally with 250  µg of propranolol (*β* adrenoreceptor blocker) 4 hours before and 6 hours after PT stroke surgery.

### Evans blue extravasation assay

To assess blood–brain barrier (BBB) permeability, Evans blue dye (2% w/v in sterile saline) was administered via intravenous injection at a volume of 100 µL per mouse 4 hours before humane killing. At 24 hours poststroke, the mice were transcardially perfused with cold phosphate-buffered saline (PBS) to remove intravascular dye before brain collection. The brains were then examined for Evans blue extravasation.

### Wire-hanging test

The wire-hanging test was performed as previously described,^11^ with slight modifications to assess motor function ability. In brief, the mice were hung on a wire stretched between two posts approximately 60 cm high. The mice were able to utilize their forelimbs to hang and balance on the wire. The time until each mouse dropped off the wire was recorded. Three hundred seconds was assigned as the maximum period, and three trials were performed for each mouse with a 5-minute resting interval between trials. The average hanging time was recorded. During the hanging wire test, if the mice reached the posts, the timer was stopped, the animal was placed back in the middle of the wire, and the timer was resumed.

### Sample collection

All samples were collected following perfusion with sterile 1x PBS in a biosafety cabinet. Sterile 1x PBS was prepared by mixing 100  mL of 10x sterile-filtered PBS (pH 7.4; Gibco, Thermo Fisher Scientific) with 900  mL of Milli-Q filtered water and then autoclaved at 121°C and 15  psi for 20 minutes. This was done to ensure that no bacterial contamination occurred in the sterile 1x PBS used for perfusion. All the equipment used for humanely killing the animals was also cleaned and autoclaved to remove any potential contamination. The mice were dipped in 70% ethanol after being euthanized. The head was decapitated, and the head was dipped in 70% ethanol again to ensure sterility. The large intestine and spleen weights, as well as the small and large intestine lengths, were recorded. The duodenum, jejunum, and ileum tissues were collected, cut in half, rolled to preserve villus morphology and fixed in 10% neutral-buffered formalin. All three regions were paraffin-embedded, sectioned and stained with periodic acid‐Schiff/Alcian blue (PAS/AB) and Masson’s trichrome via the Monash Histology Platform. The tissues used for flow cytometry analysis were harvested and used immediately. In contrast, the cecal content, the brain, and the remaining parts of the gut were snap-frozen in liquid nitrogen and stored at −80°C until needed for microbial DNA extraction or immunofluorescence.

### Infarct volume and brain edema

Frozen brains were cut into 30 mm coronal sections on a cryostat. Evenly spaced sections (separated by 210 µm for PT stroke or 420 µm for pMCAO) spanning the infarct were thaw-mounted onto poly-L-lysine-coated glass slides and stained with 0.1% thionin for infarct and brain edema analysis. Brain edema was calculated by comparing the ischemic and contralateral hemispheres using the following formula: brain edema (as a percentage of the contralateral hemisphere) = [(volume of ischemic hemisphere – volume of contralateral hemisphere)/volume of contralateral hemisphere] ∗ 100%.

### Brain and colon immunofluorescence

Serial coronal brain sections at a thickness of 10 µm were collected from the middle of the infarct for immunohistological analysis. All the sections were thaw-mounted onto 0.1% poly-L-lysine-coated glass slides and stored at −80°C. For colon sections, colon tissue was cut open along the mesenteric border, pinned flat without damaging the tissue and snap frozen in OCT (Tissue-Tek, Netherlands). All the sections were then cryosectioned at a thickness of 10 µm, thaw-mounted onto Superfrost-OT Plus glass slides (Thermo Fisher Scientific) and stored at −80°C. All immunofluorescence staining methods were performed after optimization according to the manufacturer’s protocol. Briefly, three 10 µm brain sections were air-dried before being fixed with 4% paraformaldehyde for 15 minutes. The sections were washed with 0.01 M phosphate-buffered saline (PBS, 3 × 10 minutes) and blocked with 10% goat serum (Abcam; Cambridge, USA; diluted in 0.01 M PBS) for 1 hour. The sections were then incubated with a primary antibody overnight (see Table S1), washed in 0.01 M PBS (3 × 10 minutes), and incubated with an appropriate secondary antibody at room temperature for 2 hours. The primary and secondary antibodies used are summarized in Table S1. The sections were washed in 0.01 M PBS (3 × 10 minutes) and then carefully dried before being mounted in Vectashield Antifade Mounting Medium with DAPI (Vector Laboratories, Burlingame, CA). The same protocol was used for negative controls in the absence of primary antibodies, and no immunofluorescence was detected. To ensure that no cross-reactivity occurred with single- or double-labeling immunofluorescence, controls consisted of all possible combinations of primary and secondary antibodies. For example, negative controls omitting the primary antibody were included in all immunofluorescent experiments to confirm the specificity of the staining and to rule out nonspecific secondary antibody binding. A similar protocol was used for the colon sections and fecal smears. Briefly, tissues or fecal smears were air-dried and fixed in 10% neutral-buffered formalin for 20 min, washed, labeled with primary antibody (see Table S1) overnight, washed and labeled with secondary antibody (see Table S1) for 2 h at room temperature.

### Flow cytometry

The brain, spleen, blood, mesenteric lymph nodes and gut were digested into single-cell suspensions by mechanical and/or enzymatic digestion with digestion buffer containing collagenase type XI (Sigma-Aldrich), hyaluronidase (Sigma-Aldrich), and collagenase type I-S (Sigma-Aldrich) as previously described.^10^ To obtain a single-cell suspension, samples were passed through a 70 µm cell strainer, centrifuged and resuspended in fresh FACS buffer. We then stained the single-cell suspensions with various markers, as shown in Table S2. Flow cytometry data acquisition was performed on a Fortessa X20 analyser (Becton and Dickson) at Monash FlowCore (Australia). The data were analyzed using FlowJo software version 10 according to the gating strategy in Figure S6A.

### qPCR quantification of bacteria in blood and brain

The standard for the standard curve was generated according to the manufacturer’s protocol. In brief, the bacterial 16S rRNA gene was used as the primer (F: 5’-CGGCAACGAGCGCAACCC; R: 5’-CCATTGTAGCACGTGTGTAGCC) to generate the 16S PCR product. The PCR product was then cleaned, and the concentration was measured. The number of copies per ng of DNA in the PCR product was calculated using an online tool (http://www.thermoscientificbio.com/webtools/copynumber/). We then diluted the PCR product such that the solution contained 10^5^ copies of the gene per 1 µL of DNA. To generate the standard curve for quantifying bacteria in the blood and brain, we performed a 10-fold serial dilution, starting with the 10^5^ sample. Real-time quantitative PCR (qRT‒PCR) was performed in triplicate for each sample using Fast SYBR Green Master Mix (Thermo Fisher Scientific) on a QuantStudio 7 real-time PCR system (Thermo Fisher Scientific) with one cycle of 95°C for 10 minutes, followed by 40 cycles of 95°C for 15  sec and 60°C for 60 sec.

### Microbial DNA extraction and 16S sequencing

Both the ischemic hemisphere of the brain and cecal content were collected sterilely following euthanasia and stored at −80°C as previously described. To extract bacteria from the brain, the HostZero Microbial DNA Kit (ZymoResearch) was used following the manufacturer’s protocol to first deplete the host DNA and then extract the microbial DNA. For the cecal content, we performed microbial DNA extraction using the DNeasy PowerSoil DNA isolation kit (Qiagen, Germany) according to the manufacturer’s protocol. DNA extraction was performed using brand-new, sterile, DNase- and RNase-free tubes and filtered pipette tips to prevent cross-contamination. All procedures were conducted in a laminar flow hood using autoclaved reagents and materials. The negative controls included blank tubes (no tissue) processed during DNA extraction and no-template controls for qPCR amplification. No amplification was observed in these controls, confirming the absence of detectable contamination. DNA from each sample was quantified using a Nanodrop (Thermo Fisher Scientific). Microbial DNA (10 ng/μl) was used to amplify the V4 region of the 16S ribosomal RNA, with the 515F and 806 R primers and Platinum™ Hot Start PCR Master Mix (Thermo Fisher Scientific), according to the protocol of the Earth Microbiome Project.^13^ For brain samples, all DNA extracted was used, as low numbers of bacteria were expected. A total of 240  ng of the library was pooled and purified using QIAquick PCR Purification Kit (Qiagen, Germany) before sequencing was performed at the Australian Genomic Research Facility (AGRF, Melbourne, Australia) on an Illumina MiSeq sequencer (300-bp paired-end reads).

### 16S rRNA bioinformatics analyses

The microbiome sequencing results were analyzed using QIIME2 (2020.2 version) in R Studio (version 1.2.1335).^14^ The raw reads were first trimmed to obtain at least 20 Phred quality scores, the reads were merged, and then the chimeric reads were removed. The DADA2 QIIME2 plug-in was used to remove and correct noisy reads.^15^ Alpha‐diversity metrics (ACE index, Chao1 richness index, observed species richness, and Shannon diversity index) and beta-diversity metrics (both weighted UniFrac and unweighted UniFrac) shown as principal coordinate analysis (PCoA) plots were estimated using the q2‐diversity QIIME2 plug-in after samples were rarefied for a minimum depth of 20,000 annotated sequence variants (ASVs). Data scarcity was not performed on the brain samples because of the low count number. Taxonomy bar plots at the phylum and genus levels, as well as linear discriminant analysis (LDA) effect size (LEfSe), were visualized using MicrobiomeAnalyst.^16^

### RNA isolation and qRT‒PCR

RNA from cecal tissue was extracted using TRIzol (Thermo Fisher Scientific), treated with DNase I (Thermo Fisher Scientific), and synthesized into cDNA using the High Capacity cDNA Reverse Transcription Kit (Thermo Fisher Scientific) according to the manufacturer’s protocol. qRT‒PCR was performed in duplicate for each sample using Fast SYBR Green Master Mix (Thermo Fisher Scientific) on a QuantStudio 7 real-time PCR system (Thermo Fisher Scientific) with one cycle of 95°C for 20  sec, followed by 40 cycles of 95°C for 1  sec and 60°C for 20  sec. The quantified genes and their primers are summarized in Table S3. The qRT‒PCR gene expression results were quantified using the 2(^−ΔΔCt^) method.^17^ Gene expression is expressed as the fold change relative to cecal tissue from sham‐operated animals. *β*-actin (*Actb*) was used as the reference gene.

### Statistical analyses

Quantitative data are expressed as the mean ± standard error of the mean (SEM). Statistical analyses were conducted using GraphPad Prism version 8 software (Graph-Pad Software Inc., San Diego, CA, USA). Statistical comparisons were made either by two-tailed unpaired *t* tests (when comparing two groups) or by one-way ANOVA corrected by FDR (when comparing more than two groups; two-stage step-up method of Benjamini, Krieger, and Yekutieli) or by two-way ANOVA corrected by FDR (when comparing two grouping variables; two-stage step-up method of Benjamini, Krieger, and Yekutieli). The results are expressed as the mean ± standard error of the mean (SEM). *P* < 0.05 was considered statistically significant.

## Results

### Bacteria found in the brain 24-72 hours poststroke

**Figure 1.**
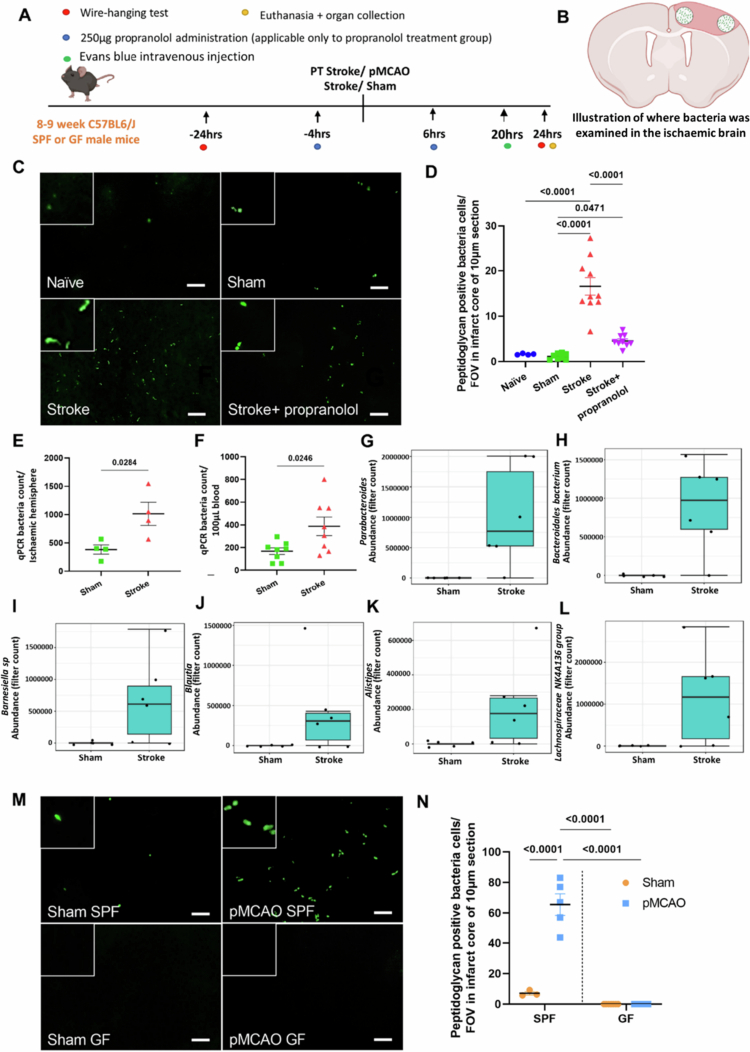
Bacteria present in the brain poststroke. (A) Experimental design of the study. (B) Illustration of where the peptidoglycan-positive bacteria were examined in the ischemic brain. Most of the bacteria were found in the infarct region circled. The number of bacteria was counted and averaged. (C) Representative images of positive peptidoglycan cells found in the ischemic hemisphere of naïve, sham, stroke and propranolol-treated mice. Scale bar: 5 µm. The inserts are magnified images of peptidoglycan-positive bacteria. (D) Quantification of peptidoglycan-positive cells in the brain infarct region 24 hours after PT stroke. Statistical test: One-way ANOVA corrected by the false discovery rate (FDR); sample size = 4–10/group. (E and F) qPCR quantification of bacteria in the ischemic right hemisphere brain and blood of PT and sham mice. Statistical test: Student’s unpaired *t* test; sample size = 4–10/group. (G–L) Increased abundances of *Parabacteroides* (*P* = 0.090), *Bacteroidales bacterium* (*P* = 0.095), *Barnesiella* sp. (*P* = 0.095), *Blautia* (*P* = 0.095), *Alistipes* (*P* = 0.095), and *Lachnospiraceae NK4A136 groups* (*P* = 0.095) were detected in the brains of poststroke mice using 16S microbiome sequencing method (*n* = 5‒6 mice/group). (M) Representative images of peptidoglycan-positive cells found in permanent middle cerebral artery occlusion (pMCAO) specific pathogen-free (SPF), sham SPF, pMCAO germ-free (GF) and sham GF mice. Scale bar: 5 µm. The inserts are magnified images of peptidoglycan-positive bacteria. (N) Quantification of peptidoglycan-positive cells in the brain infarct region 24 hours after pMCAO stroke. Statistical test: Two-way ANOVA with FDR correction. Sample size = 3–7/group. The error bars represent the mean ± SEM.

To demonstrate the presence of bacteria in the brain poststroke, we euthanized the mice 24 or 72 hours after they were subjected to photothrombotic (PT) stroke (Figure 1A) and performed immunofluorescent labeling on brain sections with a peptidoglycan antibody (a selective marker for the bacterial cell wall) (Figure 1B and Table S1). We detected numerous prokaryotic morphologies within the infarct cores of stroke mice, with little to no staining in the sham and naïve control groups (Figure 1C and 1D). Notably, the prokaryotic morphologies are typically of various shapes and sizes, ranging from 1–3 µm. Moreover, there was no evidence of peptidoglycan-immunoreactive bacteria outside of the ischemic core in the mice subjected to PT stroke, nor was there any labelling in the negative controls (data not shown). Collectively, these findings indicate that the increased presence of peptidoglycan-positive bacteria in the brain is due to the occurrence of stroke. To further verify the presence of bacteria in the brain, sections were labeled with the gram-negative bacterial marker lipopolysaccharide (LPS). Consistent with peptidoglycan immunofluorescence, LPS immunoreactivity was observed only in the infarct region and was similar in shape and size (Figure S2). Moreover, peptidoglycan labeling of bacteria in fecal samples from mice was similar to Gram staining, which further verified the bacterial labeling (Figure S2). To determine whether the bacteria are colocalized with immune cells, double immunofluorescent labeling was performed with peptidoglycan and either myeloperoxidase (a selective marker of neutrophils) or F4/80 (a marker of macrophages/microglia). All peptidoglycan-positive labels in the ischemic core did not colocalize with neutrophils or macrophages at 24 hours post-PT stroke (Figure S1). The immunohistochemical findings of bacteria present in the brain after stroke were consistent with the absolute quantification of bacteria using qPCR and 16S rRNA primers, with bacteria found in the ischemic hemisphere (Figure 1E) and blood 24 hours after PT stroke (Figure 1F). Using 16S rRNA sequencing, we identified several genera of gut bacteria within the ischemic brain post-PT stroke, including *Parabacteroides, Bacteroidales bacterium*, *Barnesiella* sp., *Blautia*, *Alistipes*, and *Lachnospiraceae NK4A136* (Figure 1G−1L). To identify whether the bacteria in the brain poststroke are endogenous, we subjected specific pathogen-free (SPF) and germ-free (GF) mice to sham or permanent middle cerebral artery occlusion (pMCAO) and performed peptidoglycan immunofluorescent labeling. Consistent with the findings from PT stroke mice, a significant number of peptidoglycan-positive bacteria were observed in the brains of pMCAO SPF mice compared to sham SPF mice. As observed in PT stroke mice, peptidoglycan-immunoreactive bacteria were found predominantly within the cerebral infarct region after pMCAO. In contrast, no positive peptidoglycan immunoreactivity was observed in the ischemic hemisphere of GF mice (Figure 1M and 1N).

### The breakdown of the blood‒brain barrier (BBB) poststroke and the role of the sympathetic nervous system (SNS) in bacterial translocation

**Figure 2.**
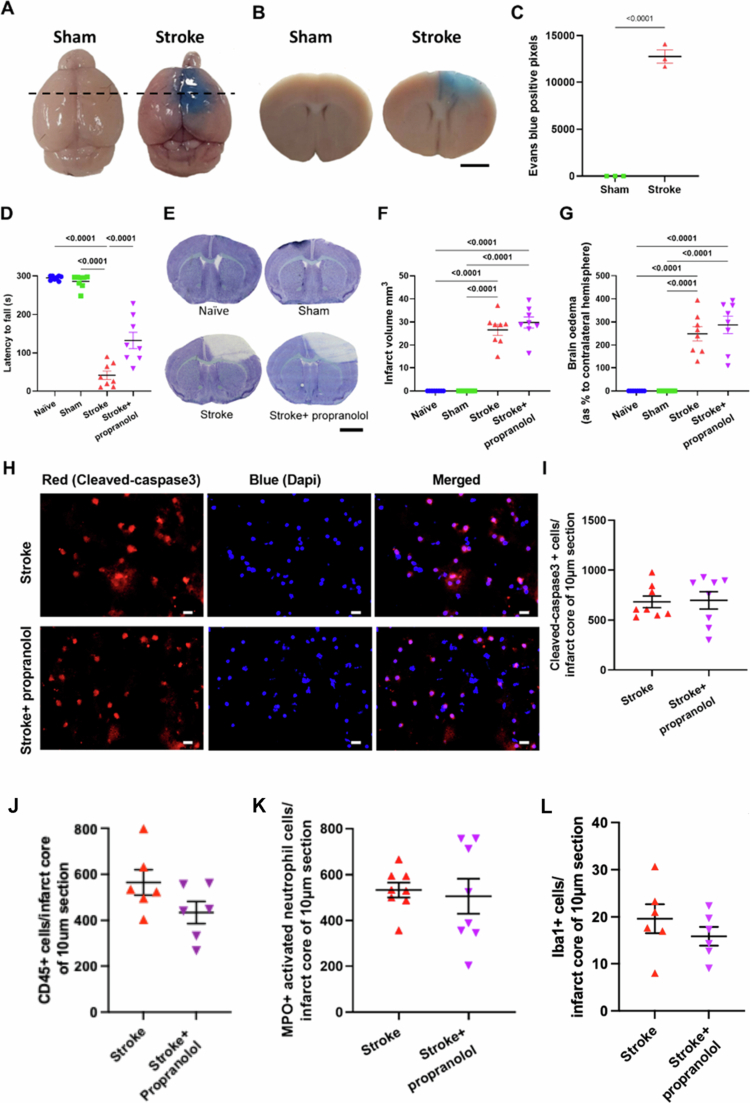
The breakdown of the blood‒brain barrier and the role of the sympathetic nervous system in bacterial translocation at 24 h poststroke. (A and B) Representative images of Evans blue dye extravasation in mice from a superior view and in corresponding coronal brain sections. The dotted lines indicate the locations of the coronal brain sections. Scale bar = 2 mm. (C) Quantification of Evans blue-positive pixels. Statistical test: Student’s unpaired *t* test. Sample size = 3/group; error bars denote the mean ± SEM. (D) Data showing improvements in motor function in the four different experimental groups, as assessed by the wire-hang test. Statistical test: One-way ANOVA with FDR correction. Sample size = 8/group; error bars denote the mean ± SEM. (E) Representative images of thionin-stained brain sections. PT stroke brain sections show the infarct region (white) and the unaffected area (purple). Scale bar: 2 mm. (F and G) Quantification of infarct volume and brain edema in naïve, sham, stroke and propranolol-treated mice measured 24 hours poststroke. Statistical test: One-way ANOVA with FDR correction. (H) Representative images of cleaved caspase-3 labeling in the infarct cores of stroke and propranolol-treated mice. Scale bar = 20 µm. (I) Quantification of cleaved caspase-3-positive apoptotic cells in the brain infarct region 24 hours after PT stroke in 10 µm sections. No significant difference was found between the stroke and propranolol-treated groups. Statistical test: Student’s unpaired *t* test. Sample size = 8/group; error bars denote the mean ± SEM. The number of (J) CD45-, (K) MPO- or (L) Iba-1-positive cells in the infarct region was quantified 24 hours poststroke in 10 µm sections. No significant difference was found between the stroke and propranolol-treated groups. Statistical test: Student’s unpaired *t* test. Sample size = 6-8/group; error bars denote the mean ± SEM.

BBB breakdown is a significant hallmark of stroke.^18^ To demonstrate the breakdown of the BBB 24 hours poststroke, we intravenously injected 2% Evans blue at 20 hours after photothrombosis (Figure 1A). At 4 hours post-injection, significant extravasation of Evans blue dye in and around the infarct core was observed in our PT model but not in the sham model, confirming BBB disruption at 24 hours poststroke (Figure 2A–2C). Previous research has shown that the SNS plays a role in bacterial translocation and that inhibiting the SNS pathway through the use of propranolol can decrease the presence of bacteria in the lung, liver, and spleen after stroke.^8^ To determine whether the translocation of bacteria to the brain involves the SNS, mice were treated 4 hours before and 6 hours after stroke with the *β*-adrenergic receptor blocker propranolol (Figure 1A). We observed a significant 2–4-fold reduction in the presence of peptidoglycan- and LPS-immunoreactive bacteria in the brain at 24 hours poststroke in the mice treated with propranolol, in comparison to PT vehicle-treated mice (Figure 1C–1D and Figure S2). In addition, propranolol-treated mice presented improved grip strength and coordination, as they were able to hang onto the wire for approximately 3-fold longer compared to the vehicle-treated mice (Figure 2D). In contrast, we did not observe any differences in infarct size (Figure 2E and 2F), brain edema (Figure 2G), the number of cleaved caspase-3-positive cells (Figure 2H and 2I), the number of leukocytes (Figure 2J), the number of neutrophils or microglia (Figure 2K and 2L), or zonula occludens-1 (ZO-1) immunoreactivity (Figure S2) in the ischemic hemisphere between vehicle- and propranolol-treated mice. Although no difference in immune cell infiltration was observed between the stroke and stroke + propranolol groups in brain sections (Figure 2 and Figure S2), this finding reflects localized analysis of the infarct core by immunofluorescence. In contrast, flow cytometry of whole-brain homogenates (Figure S5B) revealed increased immune cell presence in stroke vs. sham animals, which was consistent with a broader neuroinflammatory response. Collectively, these findings suggest that bacterial translocation poststroke is not associated with stroke-induced brain damage or immune cell infiltration.

### Propranolol treatment restores stroke-induced cellular and histological changes in the gut

**Figure 3.**
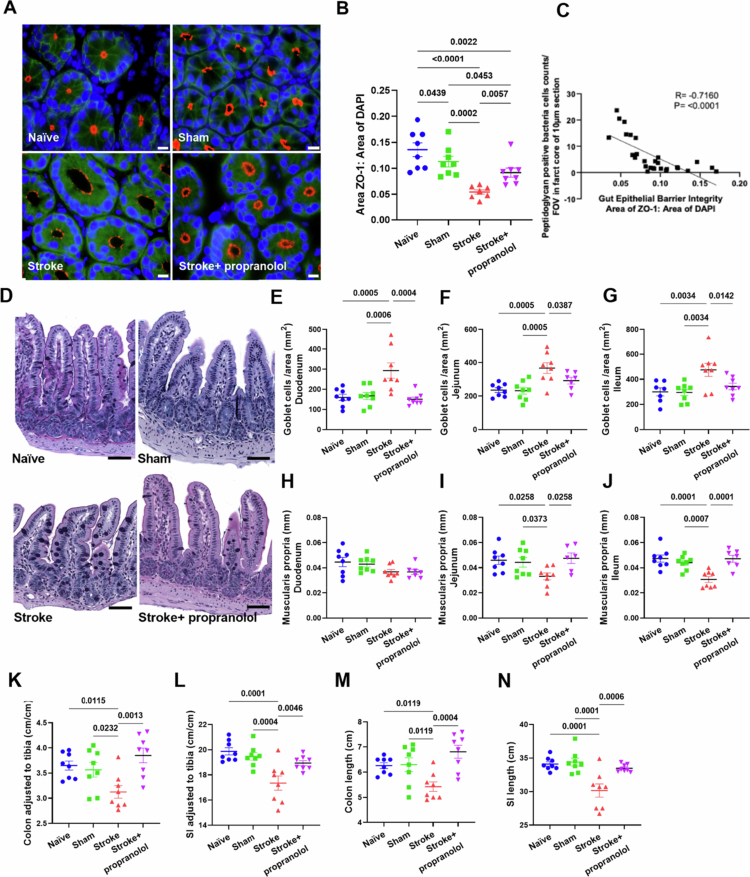
The gut-epithelial barrier is altered 24 h after stroke. (A) Representative images of zonula occludens-1 (ZO-1) labeling in the colon tissue of naïve, sham, stroke, and propranolol-treated mice. Scale bar: 10 µm. (B) Quantification of ZO-1 expression in the colon tissue of naïve, sham, stroke, and propranolol-treated mice. Statistical test: One-way ANOVA with FDR correction. Sample size = 8/group; error bars denote the mean ± SEM. (C) Graph showing the significant negative correlation between gut barrier integrity (ZO-1) and bacteria present in the ischemic brain (*r* = –0.7160, *P* < 0.0001). (D) Representative images of the ileum depicting changes in the number of goblet cells and muscularis propria thickness stained with Periodic Acid-Schiff/Alcian blue. Scale bar: 60 µm. (E, F, and G) Quantification of the number of goblet cells in the duodenum, jejunum and ileum. Statistical test: One-way ANOVA with FDR correction. Sample size = 8/group; error bars denote the mean ± SEM. (H, I, and J) Quantification of the thickness of the muscularis propria in the duodenum, jejunum and ileum regions. Statistical test: One-way ANOVA with FDR correction. Sample size = 8/group; error bars denote the mean ± SEM. (K and L) The colon and small intestine (SI) lengths were normalized to the tibia length (cm/cm). Statistical test: One-way ANOVA with FDR correction. Sample size = 8/group; error bars denote the mean ± SEM. (M and *N*) The raw colon and SI length data were measured 24 hours poststroke. Statistical test: One-way ANOVA with FDR correction. Sample size = 8/group; error bars denote the mean ± SEM.

To determine whether the gut-epithelial barrier breaks down following PT stroke, we immunolabeled colon tissue with a ZO-1 antibody, a key marker for tight junction proteins and barrier integrity. We found that the decrease in ZO-1 immunoreactivity in the colon following ischemic stroke was partially reversed by propranolol treatment (Figure 3A and 3B). We also performed a correlation analysis between gut epithelial barrier integrity scores and the number of peptidoglycan-positive bacterial cells detected in the brain and found a significant negative correlation (*r* = –0.7160, *P* < 0.0001, Figure 3C). Additionally, stroke increased the number of mucus-producing goblet cells (Figure 3D–3G) and reduced the thickness of the muscularis propria within the small intestine (with the exception of the duodenum region) (Figure 3H–3J). We also observed a significant reduction in the total length of the colon and small intestine (Figure 3K‒3N). However, propranolol treatment consistently prevented these adverse effects, resulting in a significant reduction in the number of goblet cells, an increase in the thickness of the muscularis propria, and restoration of the length of the colon and small intestine to a level comparable to those of the sham and naïve groups (Figure 3D–3N). We also detected increased expression of genes related to gut barrier integrity, such as *Cldn1*, *Tjp1*, *Tjp2* and *Ocln*, as well as inflammation, such as *Nlrp3*, *Reg3β* and *Tlr4*, in the caecum following ischemic stroke (Figure S3). However, no differences in villus length or fibrosis thickness were observed in the small intestine (Figure S4), nor were differences in organ (spleen, liver, heart, lung and kidney) or body weight (Figure S5) among the mice subjected to PT stroke and treated with either vehicle or propranolol. Moreover, propranolol did not alter ZO-1 immunoreactivity in the ischemic hemisphere 24 hours post-PT stroke (Figure S2), which suggests that BBB permeability was not altered. While we did not directly quantify the bacterial load in the blood following propranolol treatment, we observed a significant reduction in gut permeability (Figure 3B) and decreased bacterial signals in the brain (Figure 1C and 1D), alongside improved motor outcomes (Figure 2D). These findings support the interpretation that propranolol limits bacterial translocation, likely by preserving gut barrier integrity.

### Changes in immune cells in various organs

The immune system has a symbiotic relationship with the gut microbiota, including a vital role in controlling bacteria in the GI tract.^19^ To investigate the impact of stroke on the immune system, we performed flow cytometry to analyze the changes in immune cells in various organs, such as the brain, mesenteric lymph nodes, blood, spleen, and gut, following PT stroke. We used different immune cell markers (Table S2) and gating strategies, as shown in Figure S6A, to ensure accurate analysis. We found that immune cells such as microglia (CD45^int+^), inflammatory monocytes (CD45^high+^CD11b^+^Ly6C^high+^), resident monocytes (CD45^+^CD11b^+^Ly6C^low+^) and neutrophils (CD45^+^CD11b^+^Ly6C^+^Ly6G^+^) were significantly increased in the brains of poststroke mice (Figure S6B). Consistent with these findings, we observed a significant decrease in immune cells (CD45^+^) in the mesenteric lymph nodes following stroke (Figure S6C), suggesting that the mesenteric lymph nodes could be the source of immune cells within the poststroke brain. Additionally, a significant increase in neutrophils (CD45^+^CD11b^+^Ly6C^+^Ly6G^+^) was observed in the blood after stroke (Figure S6D).

### Changes in the gut microbiome poststroke

**Figure 4.**
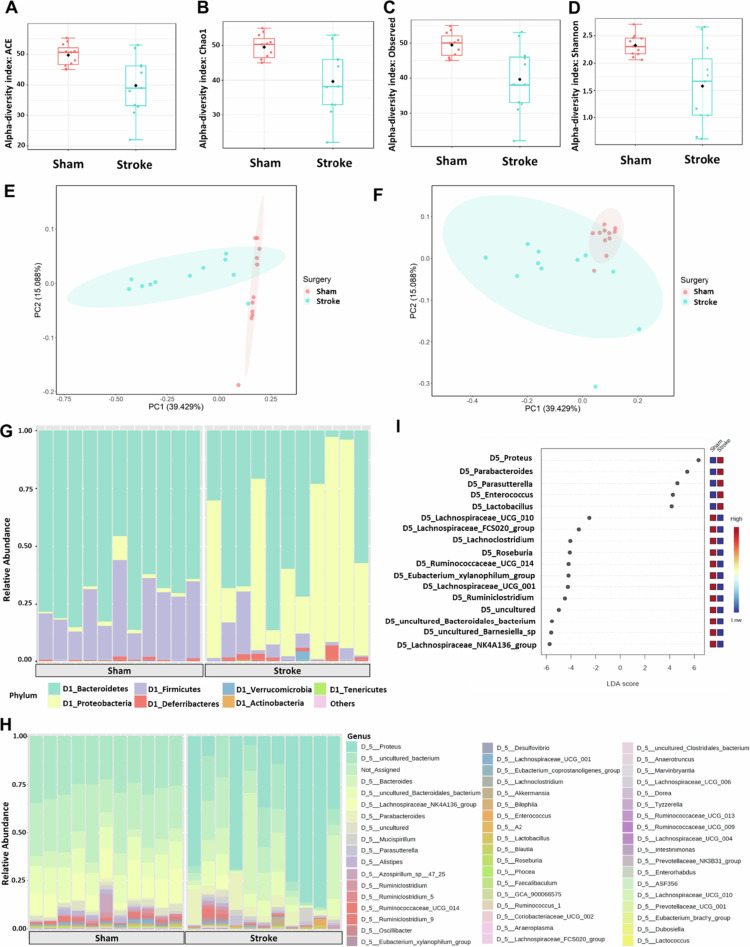
*α*- and *β*-diversity profiles of the gut microbiome 24 h after stroke. (A, B, C, and D) Box plots illustrating 95% CI *α* diversity of the gut microbiome between sham and stroke mice: ACE index (*P* value: 0.024); Chao1 richness index (*P* value: 0.020); observed species richness (*P* value: 0.022); and Shannon diversity index (*P* value: 0.011). (E and F) Principal coordinate analysis (PCoA) plots showing the clustering pattern of 95% CI *β*-diversity samples according to the type of surgery, based on weighted UniFrac distance (*P* value: 0.001) and unweighted UniFrac distance (*P* value: 0.003). (G and H) Taxa bar plots are classified at the phylum and genus levels. (I) Linear discriminant analysis (LDA) effect size (LEfSe) bar plot identifying the top significant features of stroke vs sham (threshold on the logarithmic LDA score for discriminative features: 2.0; *P* value cutoff: 0.05, FDR-adjusted). Sample size = 11/group.

To elucidate the impact of ischemic stroke on the gut microbiome, we extracted and sequenced the 16S rRNA bacterial gene. Based on the rarefaction curve (data not shown), the sampling depth set at 20,000 reads was sufficient to capture all the diversity in the samples with minimum loss. We found that the gut microbiome of PT mice had significantly lower *α* diversity, including a lower abundance-based coverage estimator (ACE) index (Figure 4A), Chao1 richness index (Figure 4B), observed species index (Figure 4C), and Shannon diversity index (Figure 4D). *β*-Diversity analyses using both weighted (Figure 4E) and unweighted UniFrac distances (Figure 4F) revealed significant differences in the microbiome composition between sham and stroke mice. There was conspicuous clustering of the gut bacterial communities in the principal coordinate analysis (PCoA) plots for the sham samples but not for the stroke samples, which was highly scattered. These results indicate that gut microbiota dysbiosis occurred 24 hours poststroke. In addition, the plot of the phylum-level taxon distribution revealed that the phylum Bacillota (formally Firmicutes; *P* = 0.002) was more abundant in sham, while Pseudomonadota (formally Proteobacteria; *P* < 0.001) was more abundant in the stroke group (Figure 4G and 4H). Specific taxa that were more abundant in sham mice included the *Eubacterium xylanophilum group* (*P* = 0.026), *Bacteroidales bacterium* (*P* = 0.006), *Barnesiella_sp* (*P* = 0.008), *Lachnoclostridium* (*P* = 0.009), *Lachnospiraceae FCS020 group* (*P* = 0.029), *Lachnospiraceae NK4A136 group* (*P* = 0.002), *Lachnospiraceae_UCG_001* (*P* = 0.026), *Lachnospiraceae_UCG_010* (*P* = 0.032), *Roseburia* (*P* = 0.002), *Ruminiclostridium* (*P* = 0.026), and *Ruminococcaceae_UCG_014* (*P* = 0.007). Conversely, *Enterococcus* (*P* = 0.010), *Lactobacillus* (*P* = 0.012), *Parabacteroides* (*P* = 0.021), *Parasutterella* (*P* = 0.032), and *Proteus* (*P* = 0.005) were more abundant in stroke mice (Figure 4I).

## Discussion

This study presents ground-breaking evidence that commensal bacteria is present in the ischemic hemisphere of two pre-clinical stroke models, and that it most likely translocates from the gut to the brain via the breakdown of the gut-epithelial barrier and is distributed via the host's blood circulation. The results also suggest that the β-blocker propranolol can prevent stroke-induced breakdown of the gut-epithelial barrier, thus reducing bacterial translocation to the brain, underscoring the significance of the gut-sympathetic tone following ischemic stroke.

Previous studies have reported the presence of bacteria in the brains of patients with neurological diseases, such as Alzheimer’s disease,^20^ Huntington’s disease^21^ and amyotrophic lateral sclerosis (ALS).^22^ While the exact role of these bacteria in the brain remains unclear, research has shown that *Chlamydophila pneumoniae,* which has been identified in the Alzheimer’s disease brain, can activate endothelial cells, producing adhesion molecules and releasing excessive cytokines,^23^ thus leading to neuronal dysfunction and cell death. While research has identified bacteria in the blood clots of stroke patients, which are thought to be derived from the oral cavity,^24^ there is no evidence to suggest that these bacteria can cross the BBB. Thus, the present study is the first to demonstrate that the bacteria typically found within the gut, including *Parabacteroides*, *Bacteroidales bacterium*, *Barnesiella* sp., *Blautia*, *Alistipes*, and *Lachnospiraceae NK4A136 group*, are located in the infarct region following ischemic stroke. Moreover, the range in sizes of peptidoglycan-positive cells further supported the presence of different bacterial genera. The possibility of bacteria being able to enter the brain was shown using Evans blue dye, which revealed that the BBB is damaged and “leaky” only in mice subjected to stroke, making it highly plausible for bacteria to cross the BBB. Notably, while increases in the levels of *Bacteroidales bacterium*, *Barnesiella sp*., and *Lachnospiraceae NK4A136* in the ischemic brain were detected, their levels were significantly lower in the gut after stroke than they were in the sham group, thus providing further evidence that the bacteria detected in the brain likely originated from the gut. Moreover, we observed no peptidoglycan-positive bacteria in the brains of germ-free mice post-MCAO, further strengthening the likelihood that the bacteria in the brain after stroke originate from the gut.

Although surprising, the low level of bacteria present in the right hemisphere of sham mice may reflect physiological microbial translocation, which has been observed in both rodents and humans, even under nonpathological conditions. Previous studies have reported bacterial DNA or peptidoglycan components in brain tissues from individuals with no acute CNS injury;^20–22,25,26^ These findings suggest that microbial components may access the brain at low levels even under baseline conditions. Nevertheless, stroke significantly increased the bacterial presence in the ischemic brain compared to sham animals, and since no bacterial signal was detected in the brains of germ-free animals, this further strengthens the specificity of the signal.

The gut barrier relies on the mucus layer, muscularis layer, and intercellular tight junctions to maintain its integrity.^27^ The current study revealed that cerebral ischaemia disrupts the gut barrier system, causing gastrointestinal inflammation that could contribute to the translocation of gut bacteria to the brain. Specifically, we observed a thinner muscularis propria, increased goblet cell secretion, and decreased expression of the tight junction protein ZO-1 following PT stroke in mice. These findings are consistent with other animal models of ischemic stroke.^8^^,^^28^^,^^29^ However, genes responsible for maintaining gut integrity were upregulated, suggesting a compensatory response to restore gut barrier function. Moreover, the upregulation of various intestinal inflammatory genes was found poststroke, indicating possible tissue damage.

Stanley et al.^8^ reported that gut bacteria can enter the circulation following stroke. Although the ischemic core is typically characterized by severely reduced or absent perfusion immediately after stroke, our Evans blue assay at 24 hours poststroke and others^11^^,^^30^ revealed dye accumulation within the infarcted region. This finding indicates significant disruption of the BBB and the presence of residual or restored perfusion sufficient to allow entry of circulating molecules. These findings suggest that translocated bacteria in the bloodstream may gain access to the peri-infarct regions as well as the infarct core at later time points. Since our imaging technique frequently detected bacterial signals within the infarct core, this finding is consistent with the permeability of Evans blue. These results support the possibility that BBB breakdown and vascular leakage enable bacterial entry into vulnerable brain regions poststroke.

It is well established that ischemic stroke activates the sympathetic nervous and immune systems, contributing to further brain injury and systemic complications.^31^ We showed an increase in all major immune cell lineages within the ischemic hemisphere post-PT stroke. It is possible that increases in both resident and infiltrating immune cells in the brain poststroke are in part due to bacterial infiltration. The observed increase in immune cells in the ischemic brain coupled with the decrease in the mesenteric lymph nodes following stroke suggests that the mesenteric lymph nodes may be the origin of some immune cells that migrate to the brain following a stroke, which is consistent with findings by Brea et al.^32^ While it is plausible that the bacteria may have accumulated within the ischemic brain owing to being trafficked via invading immune cells from the gut, our finding that peptidoglycan-immunoreactive bacteria were not co-localized with macrophages/microglia or neutrophils in the ischemic hemisphere at 24 h post-stroke suggests that this is not the case. Alternatively, it is possible that immune cells, particularly those from the gut that migrate to the brain following ischemic stoke,^32^ may do so in response to bacteria migrating to the ischemic brain.

Previously, we^8^ and others^33^ have shown that the MCAO model of ischemic stroke causes breakdown of the gut epithelial barrier and translocation of bacteria throughout the body via *β*-adrenergic signalling. To verify that this occurs in our mouse model of PT stroke and to investigate the impact of bacteria on brain damage following stroke, we used propranolol to block *β*-adrenergic signaling. We found that propranolol treatment improved gut epithelial barrier integrity by increasing ZO-1 expression in the colon and muscularis propria thickness in the small intestine and decreasing the number of goblet cells. While we did not directly assess the bacterial load in the blood following propranolol treatment, we observed a decreased bacterial signal in the ischemic brain, suggesting that sympathetic blockade may limit systemic bacterial translocation by preserving gut barrier integrity. This finding is further supported by the significant negative correlation between gut epithelial barrier integrity scores and the number of peptidoglycan-positive bacterial cells detected in the brain, which indicates that lower gut barrier integrity is associated with higher bacterial presence in the brain. Furthermore, propranolol treatment improved wire-hanging motor ability after stroke. However, we found that the reduction in bacteria in the brain was not associated with a decrease in leukocytes, neutrophils, microglia or apoptotic cells in the ischemic hemisphere. Moreover, the infarct volume was not significantly reduced in the mice treated with propranolol. While these findings differ from those reported by Monai et al.,^34^ they are consistent with outcomes reported by Prass et al.,^33^ ​​​​​who reported that propranolol pre- and poststroke does not alter cerebral infarct size. Collectively, our findings indicate that the development and severity of cerebral infarct volume and immune cell infiltration into the ischemic core after stroke are independent of bacteria within this region. While these findings do not correlate with improved functional performance, bacteria may interfere with poststroke functional recovery through mechanisms not reflected in conventional histopathological measures. For example, bacterial peptidoglycan-derived muropeptides can enter the brain and alter the activity of neurons that express Nod2, which plays an essential role in controlling appetite and body temperature.^25^ Other potential pathways include neuroimmune modulation, glial reprogramming, or altered synaptic plasticity. However, we cannot fully exclude other potential systemic effects of propranolol, such as changes in vascular tone and/or cardiac output, that may contribute to improved motor performance. Furthermore, propranolol has been shown to decrease muscle weakness and improve grip strength as well as grip endurance in patients with hyperthyroidism, which is independent of brain injury.^35^ Nevertheless, while our results indicate that there may be a link between bacterial translocation and functional impairment, we acknowledge that the current study does not establish direct causality and that further mechanistic investigation is needed to distinguish central from peripheral actions.

We acknowledge that this study has some limitations that should be considered. Our detection of bacteria in the brain provides only a static snapshot of bacteria present in that region at a specific time, as tissue analysis cannot capture the dynamics of bacterial translocation into the brain. While current whole-body imaging technology is available, its sensitivity is not optimal for detecting small numbers of translocated bacteria. Furthermore, we acknowledge the absence of direct blood bacterial measurements as a limitation and intend to address this in future studies. Culturing bacteria from blood or brain tissue would also provide further evidence of viable translocated organisms and further strengthen our findings. In addition, we examined only the presence or absence of bacteria in the brain during the acute phase of stroke. Future studies should aim to track the location of bacteria beyond this acute phase. While the current study revealed that peptidoglycan-positive bacteria are present in the brains of two different stroke models, it is crucial to understand whether the presence of bacteria in the brain poststroke is sex-, age- or species-dependent.

In conclusion, our study provides the first evidence that bacteria in the brain poststroke are likely to have migrated from the gut due to the breakdown of tight junctions in gut epithelial cells. While additional research is needed to determine whether the bacteria discovered in the brain are pathogenic or mutualistic, there is no indication that they play a role in causing acute brain damage.

## Supplementary Material

Supplemental Material.docx Supplemental Material.docx

Peh_et_al_Supplementary_file_R2 clean.docx Peh_et_al_Supplementary_file_R2 clean.docx

## Data Availability

All sequencing data were deposited in the NCBI Sequence Read Archive database (BioProject ID PRJNA960717). All other data that support the findings of this study are available from the corresponding author (BRBS) upon reasonable request.
