# Supplementary material for: Gut bacteria presence in the brain is increased after ischemic stroke in mice
Source: Gut Microbes. 2026 Jan 26;18(1):2617694. doi: 10.1080/19490976.2026.2617694 (PMC12851396; doi:10.1080/19490976.2026.2617694)
Supplement: Supplemental Material.docx [file KGMI_A_2617694_SM3815.docx]

**Gut bacteria presence in the brain is increased after ischemic stroke in mice**

**Short title:** Bacteria presence in the brain post-stroke

Alex Peh^1,2^; Evany Dinakis^1^; Michael Nakai^1^; Rikeish R. Muralitharan^1,3^; Samoda Rupasinghe^2^; Jenny L. Wilson^4^; Connie H. Y. Wong^4^; Hamdi Jama^1^; Charlotte M.O. Barker^2^; Mahnaz Modarresi^2^; Barbara K. Kemp-Harper^2^; Tenghao Zheng^1^; Francine Z. Marques^1,5*^; Brad R.S. Broughton^2*^

^1^Hypertension Research Laboratory, School of Biological Sciences, Monash University, Melbourne, Australia

^2^Cardiovascular & Pulmonary Pharmacology Group, Department of Pharmacology, Monash University, Melbourne, Australia

^3^Institute for Medical Research, Ministry of Health Malaysia, Kuala Lumpur, Malaysia

^4^Centre for Inflammatory Diseases, Department of Medicine, School of Clinical Sciences at Monash Health, Monash Medical Centre, Clayton, Victoria, Australia

^5^Heart Failure Research Group, Baker Heart and Diabetes Institute, Melbourne, Australia

*Contributed equally as senior authors

**Correspondence to**: A/Prof Brad Broughton. Address: Building 13E, 9 Ancora Imparo Way, Monash University, Victoria Australia 3800. E: bradley.broughton@monash.edu

**Supplementary Figures**

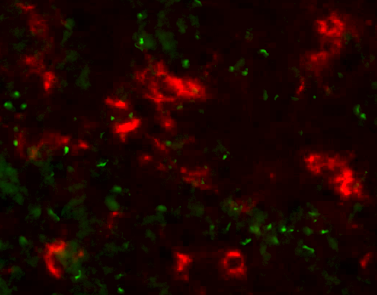


**A**


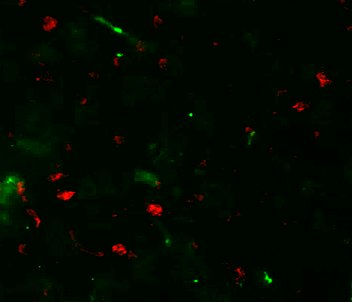


**B**

**C**

**72 h Post-Stroke**

P<0.001

**Supplementary Figure 1| Bacteria and immune cells in the brain post-stroke.**

**A-B** Representative images showing peptidoglycan-positive cells (A-B, green) are not co-localised with F4/80 (A, red) or MPO (B, red) immunoreactive cells in the cerebral infarct 24 h after PT stroke. Scale bar: 20µm. **C** Quantification of peptidoglycan-positive cells in the brain infarct region 72 hours after PT stroke.

**
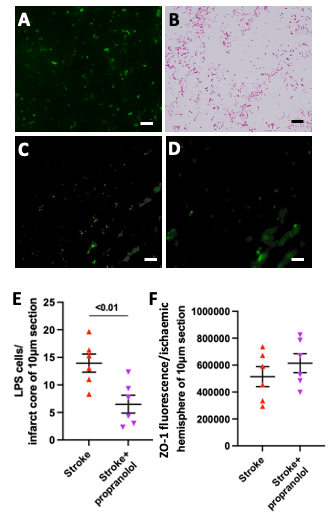
**

**Supplementary Figure 2| Peptidoglycan and LPS labelling as well as zonula occludens-1 (ZO-1) immunoreactivity in the brain post-stroke.**

Representative images of (A) peptidoglycan immunoreactivity and (B) gram staining in faecal smears. Scale bar: 15 μm. Representative images of (C-D) LPS immunoreactivity in the ischaemic hemisphere of mice treated with (C) vehicle or (D) propranolol. Scale bar: 15 mm. Quantification of (E) LPS or ZO-1 (F)-positive immunoreactivity in the brain infarct region of mice treated with vehicle or propranolol. In mice treated with propranolol, LPS immunoreactive cells were reduced in the infarct, but IBA-1 and ZO-1 labelling was not altered. Statistical test: Student’s unpaired t-test. Sample size= 6/group; error bars denote mean±SEM.

**
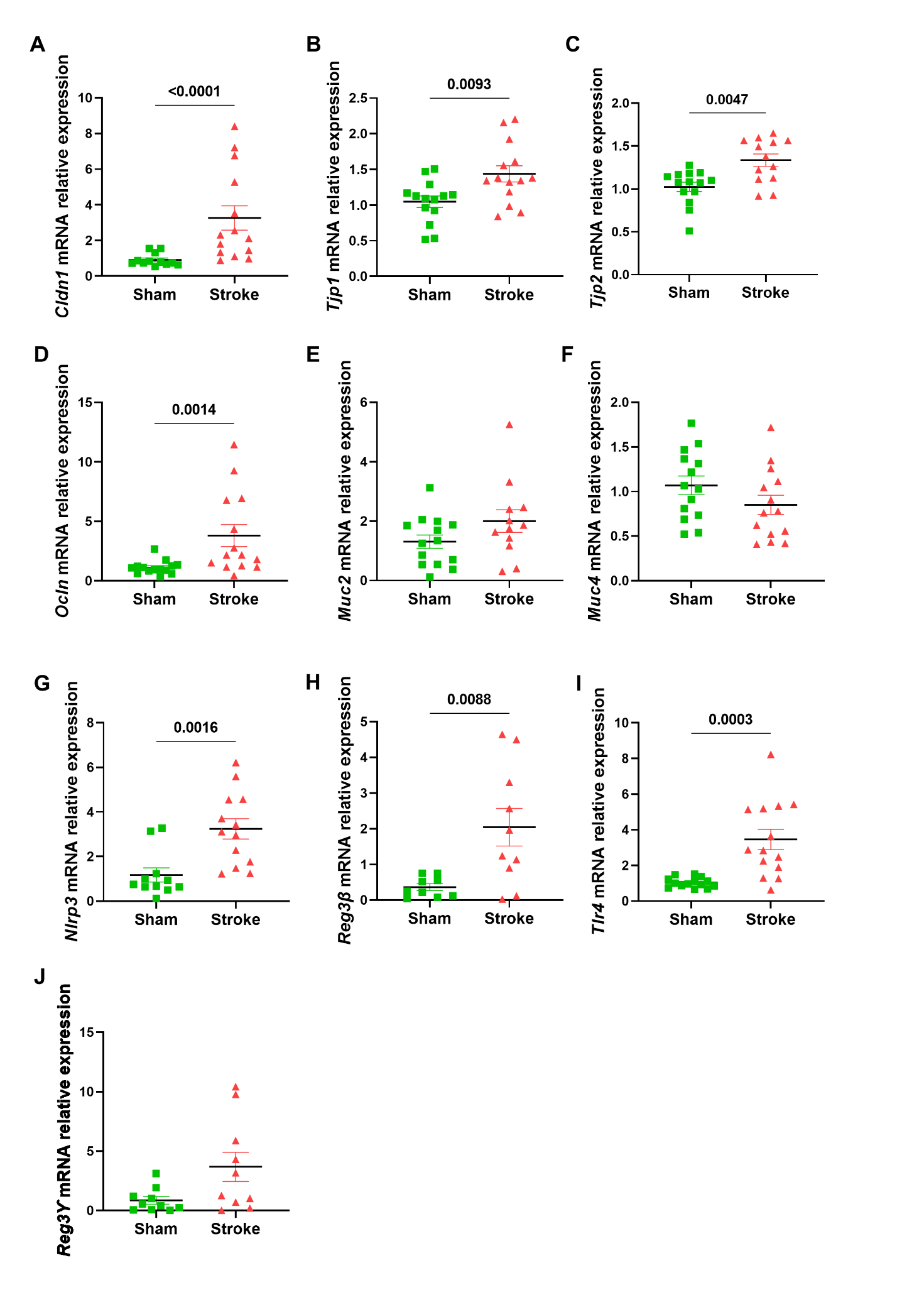
**

**Supplementary Figure 3| qPCR quantification of gene expression in caecal tissue.**

**A – J** The genes expression of gut epithelial integrity: *Cldn1,* *Tjp1, Tjp2, Ocln, Muc2, Muc4*, and inflammation: *Nlrp3,* *Reg3β, Tlr4, Reg3γ* of sham and PT mice were performed. Statistical analysis: Two-tail unpaired-t test. Sample size= 9-14/group; error bars denote mean±SEM.

**
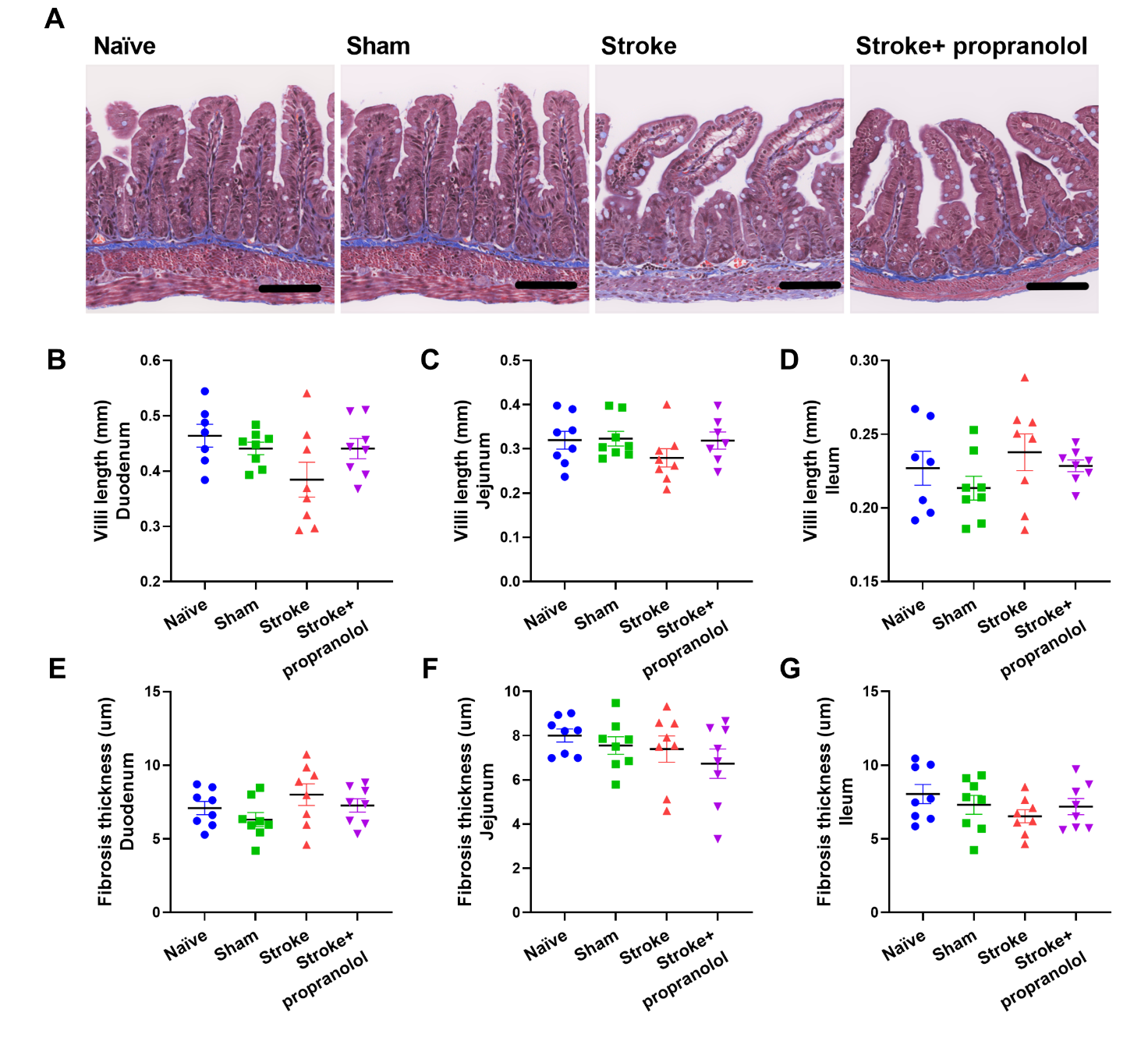
**

**Supplementary Figure 4| Villi length and fibrosis thickness between sham and stroke mice.**

**A** Representative images showing the fibrosis thickness (region in blue colour) and villi length in naïve, sham, stroke and propranolol-treated mice. Scale bar: 100µm.

**B-D** Quantification of the villi length in the duodenum, jejunum, and ileum region, respectively. Statistical test: One-way ANOVA corrected with FDR. Sample size= 8/group; error bars denote mean±SEM.

**E-G** Quantification of the fibrosis thickness in the duodenum, jejunum and ileum region, respectively. Statistical test: One-way ANOVA corrected with FDR. Sample size= 8/group; error bar denotes mean±SEM.

**
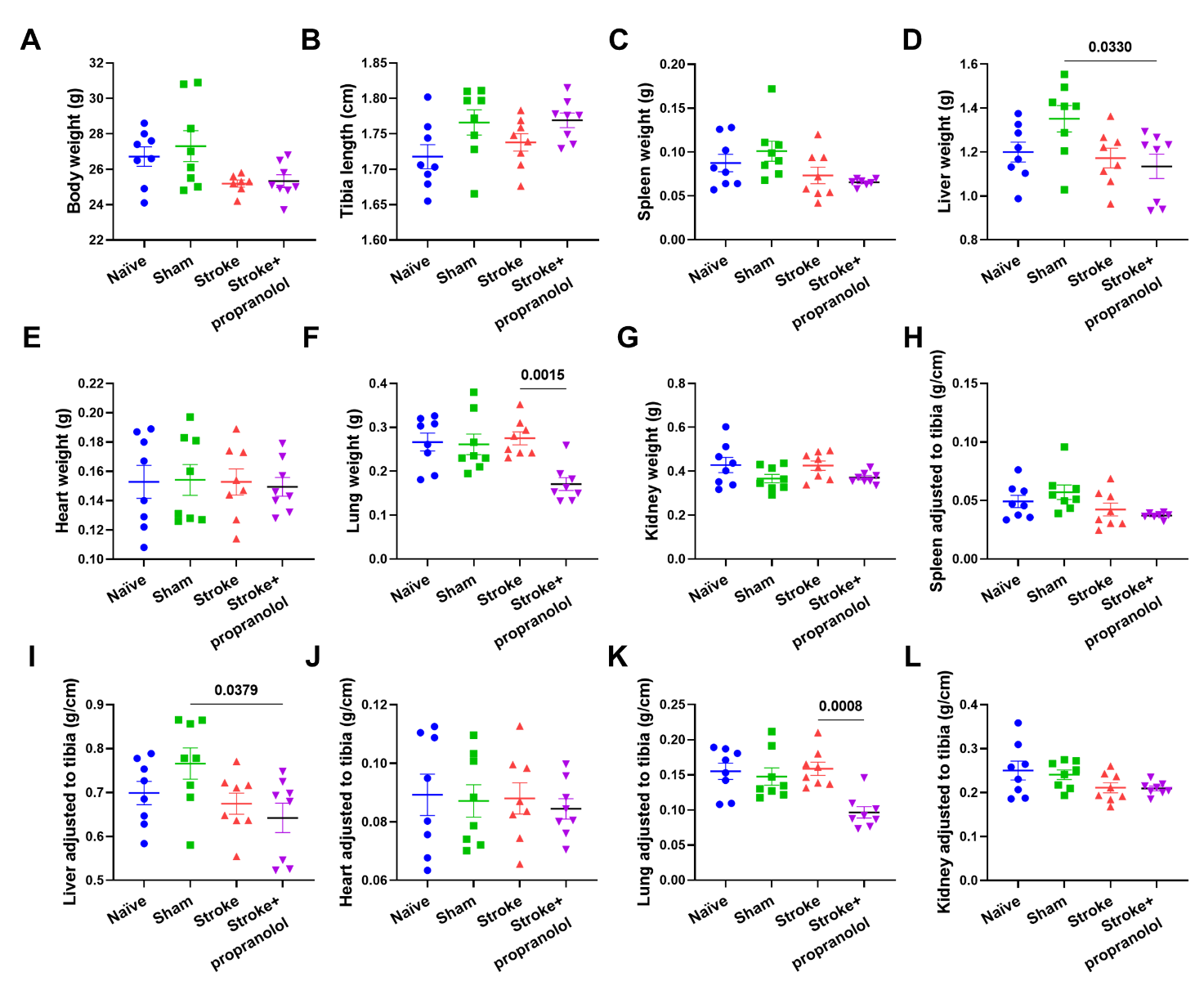
**

**Supplementary Figure 5| Body and organ weights between groups.**

**A-G** The body weight, tibia length and raw weight of spleen, liver, heart, lung, and kidney was measured 24-hours after surgery. Statistical test: One-way ANOVA corrected with FDR. Sample size= 8/group; error bars denote mean±SEM.

**H-L** The adjusted weights to tibia length of spleen, liver, heart, lung, and kidney was calculated. Statistical test: One-way ANOVA corrected with FDR. Sample size= 8/group; error bars denote mean±SEM.


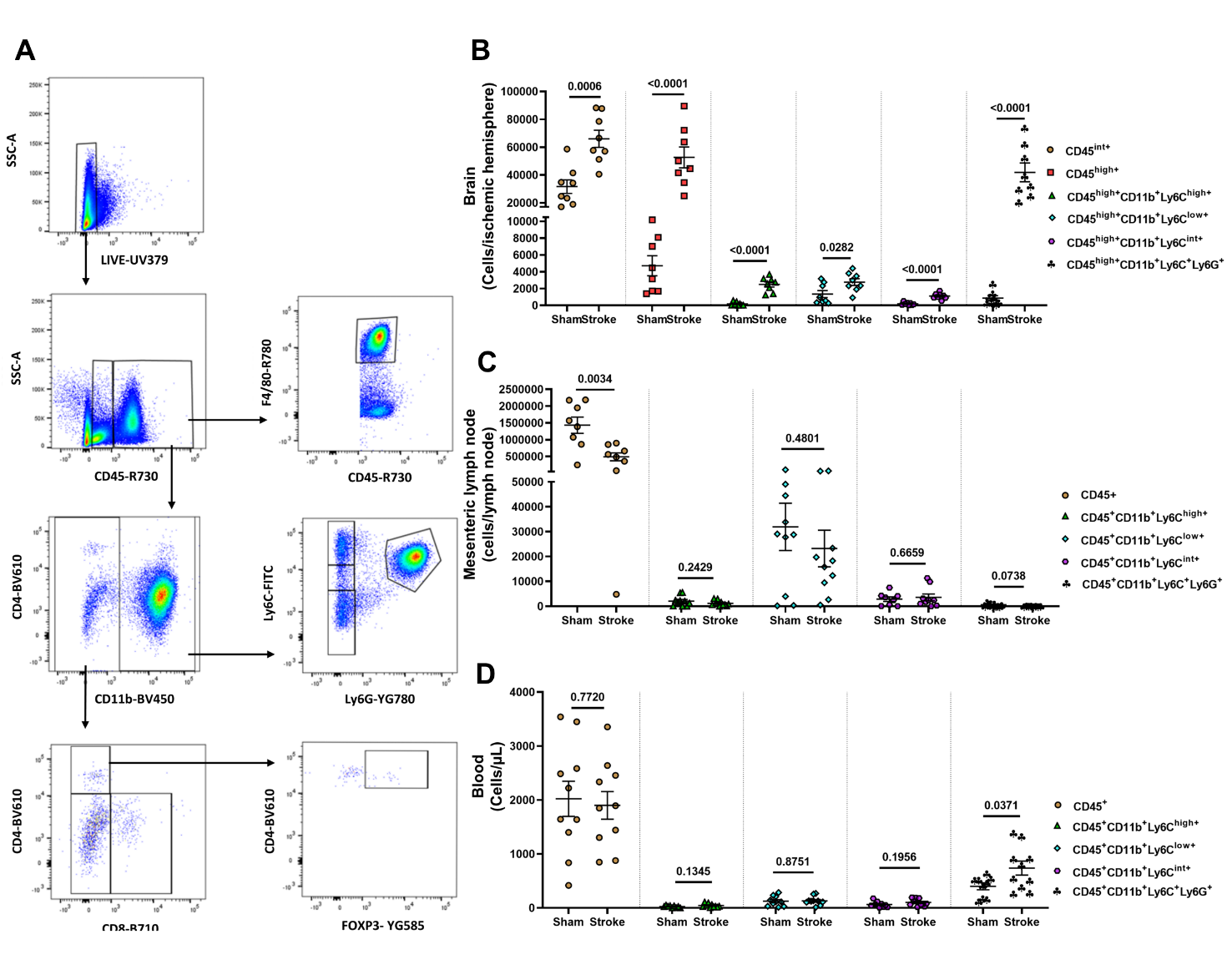


**Supplementary Figure 6| Gating strategy for immune cell populations and changes in immune cell counts of various organs following stroke.**

**A** Flow cytometry data was analysed according to the gating strategy. The gating strategy was similar for other organs except, gated on CD45^+^ instead of CD45^high+^. No CD45^int+^ and CD45^high+^ populations in other organs.

**B-D** Immune cell infiltration was assessed in the brain; mesenteric lymph node and blood between sham and stroke mice 24-hours following surgery using flow cytometry. Statistical test: Student’s unpaired t-test. Sample size= 8-14/group; error bar denotes mean±SEM. Microglia (CD45^int+^); Inflammatory monocytes (CD45^+^CD11b^+^Ly6C^high+^); resident monocytes (CD45^+^CD11b^+^Ly6C^low+^); neutrophils (CD45^+^CD11b^+^Ly6C^+^Ly6G^+^).

**Supplementary Tables**

Table S1: List of primary and secondary antibodies used for immunofluorescence.

| Tissue | Primary antibody | Dilution | Incubation temperature | Secondary antibody | Dilution |
| --- | --- | --- | --- | --- | --- |
| Brain | *Mouse anti-bacterial peptidoglycan (MAB995)* | 1:100 | Room temperature | Mouse on mouse Immunodetection Kit, Fluorescein (FMK-2201) | According to the manufacturer’s protocol |
|  | *Rabbit anti-MPO (ab9535)* | 1:200 | Room temperature | Goat anti-rabbit IgG Alexa Fluor 594 (Life Technologies) | 1:500 |
|  | Rabbit anti-Cleaved Caspase-3 (Asp175) (mAb9664) | 1:500 | 4˚C | Goat anti-rabbit IgG Alexa Fluor 594 (Life Technologies) | 1:500 |
|  | Rat F4/80 (MCA497R) | 1:100 | Room temperature | Goat anti-rabbit IgG Alexa Fluor 594 (Life Technologies) | 1:500 |
|  | Mouse LPS (C6) (MA5-41631) | 1:200 | Room temperature | Mouse on mouse Immunodetection Kit, Fluorescein (FMK-2201) | According to the manufacturer’s protocol |
|  | Rabbit Anti-Iba1 (EPR16588) | 1:200 | Room temperature | Goat anti-rabbit IgG Alexa Fluor 594 (Life Technologies) | 1:500 |
|  | Mouse anti CD45 (14045182) | 1:200 | Room temperature | Mouse on mouse Immunodetection Kit, Fluorescein (FMK-2201) | According to the manufacturer’s protocol |
| Colon (gut) | *Rabbit anti-mouse ZO-1 (61-7300)* | 1:100 | 4^o^C | Goat anti-rabbit IgG Alexa Fluor 594 (Life Technologies) | 1:500 |
|  | *Rat anti-mouse CD32/EpCam (14-5791-81)* | 1:500 | 4^o^C | Goat anti-rat IgG Alexa Fluor 488 (Life Technologies) | 1:500 |

EpCam, epithelial cellular adhesion molecule; MPO, myeloperoxidase; ZO-1, zonula occludens-1

Table S2: List of markers used for flow cytometry.

| **Markers** | **Concentration** | **Conjugate** | **Fluorochrome** | **Clone** | **Manufacturer** |
| --- | --- | --- | --- | --- | --- |
| CD45 | 1/500 | AF700 | R730 | 30-F11 | Bioleg |
| Ly-6G | 1/1000 | PECy7 | YG780 | 1A8 | Bioleg |
| Ly6C | 1/500 | FITC | B530 | HK1.4 | Bioleg |
| F4/80 | 1/500 | APC-Cy7 | R780 | BM8 | Bioleg |

AF, Alexa Fluor; APC, Allophycocyanin; BV, Brilliant Violet; Cy, Cyanine; FITC, Fluorescein isothiocyanate; PE, Phycoerythrin; PerCP, Peridinin-Chlorophyll-Protein

Table S3: A list of primer sets used for qRT-PCR.

| Pathway target | Gene name | Primer sequences |
| --- | --- | --- |
| Gut epithelial integrity | Claudin 1 (Cldn1) | F: 5’- AAT TTC AGG TCT GGC GAC ATT |
|  |  | R: 5’- GGG GTC AAG GGG TCA TAG AA |
|  | Mucin 2 (Muc2) | F: 5’- GCC CAC CTC ACA AGC AGT AT |
|  |  | R: 5’- GTC ATA GCC AGG GGC AAA CT |
|  | Mucin 4 (Muc4) | F: 5’- TCC TCT TGC TAC CTG ATG CTC T |
|  |  | R: 5’- GCT CAT TTG GGA TGT TCT GGT G |
|  | Tight junction protein 1 (Tjp1) | F: 5’- GGG CTC CTG GGT TTG GAT TT |
|  |  | R: 5’- GCA ACT CGG TCA TTT TCC TGT A |
|  | Tight junction protein 2 (Tjp2) | F: 5’- AAA GCA GAG CCG AGC AAA TGG |
|  |  | R: 5’- GCT CTT GCG GAG GTT CTT CT |
|  | Occludin (Ocln) | F: 5’- TTG AAC TGT GGA TTG GCA GC |
|  |  | R: 5’- AAG ATA AGC GAA CCT TGG CG |
| Inflammation | NLR family pyrin domain containing 3 (Nlrp3) | F: 5’- GGT GAC TTT GTA TAT GCG TGT TCT |
|  |  | R: 5’- GGG CTT AGG TCC ACA CAG AAA |
|  | regenerating islet-derived protein 3β (Reg3β) | F: 5’- GGA AGA CAG ACA AGA TGC TGC |
|  |  | R: 5’- CTA ATG CGT GCG GAG GGT AT |
|  | regenerating islet-derived protein 3ϒ (Reg3ϒ) | F: 5’- AGG ACA TCT TGT GTC TGT GCT |
|  |  | R: 5’- TCA TAG CCC AGT GTC GGG T |
|  | Toll-like receptor 4 (Tlr4) | F: 5’- GGT AAG GTT GTC TTG ACG GAA C |
|  |  | R: 5’- GCC TCA GAG AAG GTA TCC AAC A |
| Housekeeping | β-actin (Actb) | F: 5’- AAC GGC TCC GGC ATG TGC AAA G |
|  |  | R: 5’-ATC ACA CCC TGG TGC CTA GGG CG |
